# Experimental Methodology for the Separation Materials in the Recycling Process of Silicon Photovoltaic Panels

**DOI:** 10.3390/ma14030581

**Published:** 2021-01-27

**Authors:** Ines Riech, Carlos Castro-Montalvo, Loïs Wittersheim, Germán Giácoman-Vallejos, Avel González-Sánchez, Cinthia Gamboa-Loira, Milenis Acosta, José Méndez-Gamboa

**Affiliations:** 1 Laboratorio de Ciencia de Materiales, Facultad de Ingeniería, Universidad Autónoma de Yucatán, Mérida 97130, Mexico; cacm_91@hotmail.com (C.C.-M.); lois.wittersheim@eleves.ec-nantes.fr (L.W.); adiaz@correo.uady.mx (M.A.); jmendez@correo.uady.mx (J.M.-G.); 2 Laboratorio de Ingeniería Ambiental, Facultad de Ingeniería, Universidad Autónoma de Yucatán, Mérida 97130, Mexico; giacoman@correo.uady.mx (G.G.-V.); avel.gonzalez@correo.uady.mx (A.G.-S.); cinthia.gamboa@correo.uady.mx (C.G.-L.)

**Keywords:** photovoltaic panels, recycling, etching, crystalline silicon, metal separation

## Abstract

As the use of photovoltaic installations becomes extensive, it is necessary to look for recycling processes that mitigate the environmental impact of damaged or end-of-life photovoltaic panels. There is no single path for recycling silicon panels, some works focus on recovering the reusable silicon wafers, others recover the silicon and metals contained in the panel. In the last few years, silicon solar cells are thinner, and it becomes more difficult to separate them from the glass, so the trend is towards the recovery of silicon. In this paper, we investigate the experimental conditions to delaminate and recovery silicon in the recycling process, using a combination of mechanical, thermal, and chemical methods. The conditions of thermal treatment to remove the ethylene-vinyl acetate (EVA) layer were optimized to 30 min at 650 °C in the furnace. To separate silicon and metals, the composition of HF/HNO_3_ solution and the immersion time were adjusted considering environmental aspects and cost. Under the selected conditions, panels from different manufacturers were tested, obtaining similar yields of recovered silicon but differences in the metal concentrations.

## 1. Introduction

The development of solar photovoltaic (PV) energy is linked to the generation of photovoltaic (PV) waste once the PV systems reach the end of their life, so the solar photovoltaic industry to minimize this negative impact must work out in environmentally sustainable practices. The total cumulative installed capacity for PV in the world at the end of 2019 reached 627 GW [1]. Silicon-based solar modules are the dominant technology, with approximately 95% of the global PV module market [2]. The higher efficiency and the continuous reduction in their costs make these modules more competitive than other materials. However, some issues must be addressed to improve this technology, one of them is to mitigate its environmental effects [3,4]. It is well known that recycling PV waste is crucial, not only to avoid environmental pollution but also to refrain from depleting the planet’s mineral resources.

A silicon photovoltaic module is composed of an aluminum frame, glass, ethylene-vinyl acetate (EVA), silicon cells, metallic connectors (copper, silver, lead), and a polymer backsheet as Tedlar and Polyethylene Terephthalte (PET) in most cases [5]. The most important material in PV modules is silicon since it is highly required and represent approximately half the total module cost [6]. It is included in the list of critical raw materials for the European Union [7]. Besides, precious metals such as silver also have high demand nowadays, and their market price increases because of their low availability. Silver is present in conductive pastes used in the contact lines and represents less than 0.1% of the total weight of the panel [8]. The mining of this precious metal implies a negative environmental impact, so the use of secondary raw material obtained by other methods as recycling becomes a very important issue. Another relevant material used in PV modules from the point of view of environmental damage is lead, which is present in the alloy Sn/Pb used as solders for solar cell interconnections. This metal stays in PV waste and can be dispersed into the environment and harm human health [9]. For that reason, it is important to identify and separate it to dispose safely.

Different recycling processes for silicon-based modules have been reported over the past two decades, which in general combine two of these methods in different stages: mechanical, thermal, and chemical treatment. The mechanical methods include crushing, attrition, and vibration for glass separation and is the less polluting method compared to the other two [10,11,12]. Thermal treatment is mainly used to remove the polymeric fraction of the photovoltaic panel, i.e., EVA resin and backsheet materials [13,14]. This is one of the steps that demands more energy and produces higher environmental contamination due to the emission of toxic gases [15,16]. The chemical process is applied to recover silicon and metals by dissolution in acid or basic solutions [5,11,14,17]. The separation efficiency of different layers is high but, in some cases, they use toxic reagents that can hardly be implemented on a larger scale production. Consequently, the researcher working on the recycling of photovoltaic panels, focus their attention on achieving a high efficiency process, considering the impact on human health, and the ecosystem [18]. The three treatment methods have been applied in the same process, as is the case of Pagnanelli et al. who reported a process that combines crushing and thermal treatment followed by chemical treatment to recover fragments of glass and metals from different kinds of panels [12] or the Full Recovery End of Life Photovoltaic (FRELP) process developed at a pilot scale, based on a sequence of mechanical and thermal treatments followed by acid leaching and electrolysis [15]. The alternative procedure was investigated, using thermal treatment to remove EVA resin followed by chemical etching to obtain the silicon in a wafer form with the required purity level [5]. Other authors have used organic solvent to separate tempered glass [19], or to recover undamaged silicon solar cells [20]. All methods have advantages and disadvantages that must be properly considered.

Although the general structure of silicon PV modules is the same, different manufacturers use different procedures and raw materials, such as antireflection coatings (AR), encapsulating polymer films, backsheets, and metal content. These differences are also made more drastic due to the manufacturing date of the modules. Most of the reported data about the recycling process concerns modules manufactured decades ago, whose composition may be different from modern ones’ [7]. This heterogeneity about the composition of PV modules represents a limitation; in particular, the efficiency of the metal recovery after chemical treatment depends on the amount and nature of these materials in the PV module. Solar cells from different manufacturers need treatments specifically adapted to a given structure and composition [5].

The PV industry in Mexico is modest, but in the last year, Mexico appears among the countries with the fastest growth in the installed capacity of photovoltaic panels in America, behind the USA and Brazil. Nowadays, many panels are being installed and the outlook is that soon the installed capacity will grow exponentially [1,21]. In Mexico, the solar industry is still young, and since PV modules have not reached the end of their life, their recycling is not an issue yet. However, according to the estimated growth of PV waste in the future, around 2045 Mexico will have 690,907 metric tons of PV waste [22], so it is necessary to plan a recycling industry considering the photovoltaic technologies that are currently being installed and that will be the modules to be recycled in the future. Additionally, the approach that should be taken for the recycling process in Mexico is to recover the silicon, not the silicon wafer. This is because the modern cells are thinner and the risk of cracking increases in the process, so the recovery of silicon particles is more convenient [6]. In the present work, we describe the optimization of a lab-scale methodology using mechanical, thermal, and chemical method. This procedure was applied to damaged silicon modules that are currently installed from different manufacturers in Mexico.

## 2. Materials and Methods

Two polycrystalline panels, series PLM270 and PLM260 from Perlight company (Wenling, China)) (samples P1 and P2), and one monocrystalline from the Yingli company (Tianjin, China) (sample P3), were used for test purposes. First, the aluminum frames and junction box were removed manually. The modules were cut into pieces with an approximate size of 10 × 10 cm^2^, which underwent the separation of glass and backsheet from the remaining materials. Thereafter, were prepared samples of 2 × 2 cm^2^ (weighing approximately 1 g) to undergo chemical treatment.

In order to remove the glass, which was fragmented but still glued to modules, a quartz halogen lamp (Argos, 9400450 model, Argoselectrica, Mexico) was used to soften the encapsulating EVA, allowing manual separation of the glass. Before starting to remove the glass pieces, the softening temperature of the EVA must be reached, which ranges between 90 and 120 °C. The obtained flexible sheets composed of solar cells, metallic connections, and polymeric parts were scraped to remove the backsheet. In the following stage, thermal treatment was used to eliminate the remaining organic material, i.e., EVA, the encapsulating layers. For this purpose, we used a furnace Barnstead Thermolyne F6020C model. The samples were weighed before and after incineration to establish the weight loss. In the last stage, the resultant ash was dissolved in a leaching process to separate silicon and metals. Before carrying out this process, an experiment to evaluate the immersion time necessary to obtain the largest amount of silicon and metals was conducted. A total of 15 samples from the polycrystalline panel, P1 were separated into five groups. They were dipped in the solution for different periods at room temperature. Once the optimal time was found, the other samples of panels P2 and P3 were treated with these conditions. After acid etching, the solutions were filtrated to obtain solid silicon particles.

Statistical differences between silicon, silver, copper, and lead recoveries, were evaluated per time of exposure using one-way Analysis of variance (ANOVA) test and Fisher’s Least Significant Difference (LSD) in Statgraphics plus (2010) statistical software (Statpoint Technologies, Inc., Warrenton, VA, USA. The significance in all statistical analyses was established at < 0.05.

The chemical composition of the filtrated product was obtained using Energy Dispersive X-ray Spectroscopy (EDX), (JEOL model JSM-7601F, Tokio, Japan). The resulting solution was analyzed by Atomic Absorption Spectroscopy technique (Varian model AA240 FS, Varian, CA, USA) to quantify Cu, Ag, and Pb.

## 3. Results and Discussion

### 3.1. Thermal Treatment

**Figure 1:**
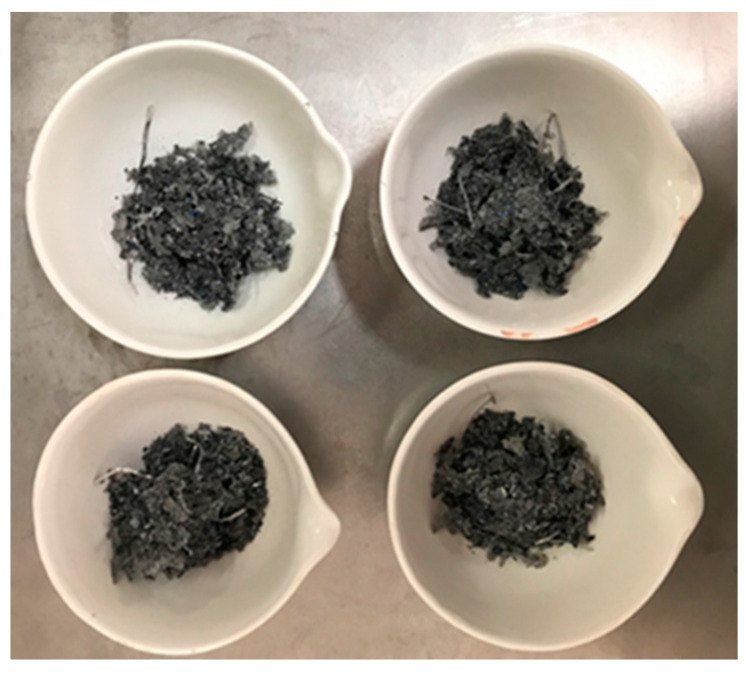
Samples after removing glass and backsheet.

After removing the glass and backsheet manually, the samples appeared as shown in Figure 1. The next step in the recycling process was the removal of EVA layers, which can be performed by two methods, chemical or thermal. According to literature reports, thermal separation is the more promising alternative from an economic and ecological point of view [5,23]. We found different reported conditions to complete EVA degradation. Some authors state that at least 1 h treatment at 500 °C is required [24,25,26]. However, other authors reported that EVA was completely removed at 600 °C for 1 h [4,12,19]. In this work we tested combinations of different times, from 5 to 60 min, and temperatures from 400 to 650 °C to achieve complete combustion. The criterion for determining whether the combustion was complete or not was the metallic blue color of the product. The presence of dark matter in the product indicates that EVA and his derived hydrocarbons were not eliminated from the samples, this combination of parameters was classified as not suitable. Our results confirmed that after 1 h at 550 °C the combustion of EVA was achieved. If the treatment time or the temperature rose above these values, it did not change the results, but the energy consumption increased. We made a rough estimation of the energy consumption, and determined that 30 min in the furnace at 650 °C were the optimal conditions to guarantee the complete combustion of the polymeric material spending less energy. The product after the thermal process was blue metallic powder.

### 3.2. Chemical Treatment

#### 3.2.1. Determination of Chemical Treatment Conditions

The recovered product after removing EVA contained materials used for the antireflective coating, the p-n junction, and the contact electrodes, in addition to silicon. To recover silicon, we performed a chemical etching process. The parameters that must be controlled during this treatment were the composition and concentration of the acid solution, plus the temperature and the time to which the samples are subjected to the process. The use of high temperatures requires energy expenditure, and an excess of reagents increases the possibility of environmental contamination and cost; so, it was desirable to minimize these parameters.

Several mixtures were tested to recover silicon, but their efficiency depended on the composition of PV technology [5]. In this work, the composition of etching solution was adjusted to 5 mL of HNO_3_ (70%) and 0.5 mL of HF (40%), in a 10:1 proportion. The chemical treatment was carried out at room temperature. Using the equations of the reactions of nitric acid and hydrofluoric acid on the metals and silicon surface layers, it is possible to calculate roughly the minimum quantity of acid to etch all the materials contained in the metallic contacts and the silicon cells. First, regarding nitric acid HNO_3_, the oxidations of copper, lead, and silver by this reagent were considered separately, to calculate the minimum amount necessary to dissolve the whole metallic solid and create the ions in solution, respectively Cu^2+^, Pb^2+^, and Ag^+^. The aim was to obtain, at the end of the reaction, a positive value for the quantity of acid, and zero for the quantity of metal. The highest value recorded by spectroscopy for each metal is used as the initial quantity to dissolve, namely 0.1348 g, 0.0101 g, and 0.0217 g for Cu, Pb, and Ag respectively. A minimum quantity of acid for each of these reactions is obtained, and after adding these values, a minimum quantity for the three metals is calculated. Because the formation of complex ions due to the presence of hydrofluoric acid and various metallic ions in solution is not considered here, it is necessary to use a margin, and double this value. The quantity of acid used in the experiments, 5 mL, was more than enough, but it would be ideal to include the influence of the different reactions on each other to calculate more accurately the minimum quantity of acid, and hence to minimize the chemical pollution.

The hydrofluoric acid promoted complete etching of the metals, due to its high aggressivity and possibility to dissolve almost all inorganic materials, except silver. It dissolved the oxidation layers that could form because of the action of nitric acid on metals, and the layers of the silicon bits, formed of a regular oxide SiO_2_ and an anti-reflective layer, probably Si_3_N_4_, according to Szlufcik [27]. Making the same assumption as in the previous paragraph, that the reaction of these silicon compounds with HF can be considered separately and summing up the values to obtain a total amount of acid necessary to etch both entities, a minimum quantity is found. The volume used experimentally should suffice for this purpose, and the progressive discoloration of the silicon bits could indicate the removal of the surface layers.

After the ashes of the combustion process were obtained, the time required to immerse the samples in the solution and achieve the greatest dissolution of the metals was investigated experimentally. The five groups of ashes from module P1 were separated and treated in the solution during different immersion times: 1.5, 3, 6, 12, and 24 h. In this leaching process, the metals were transferred to the solution while the silicon bits were deprived of their surface layers and appeared as a dark powder. After the solution was filtered, it was neutralized with boric acid, to diminish the corrosivity of the HF acid, and diluted with distilled water to obtain 100 mL of the ion complex solution.

**Figure 2:**
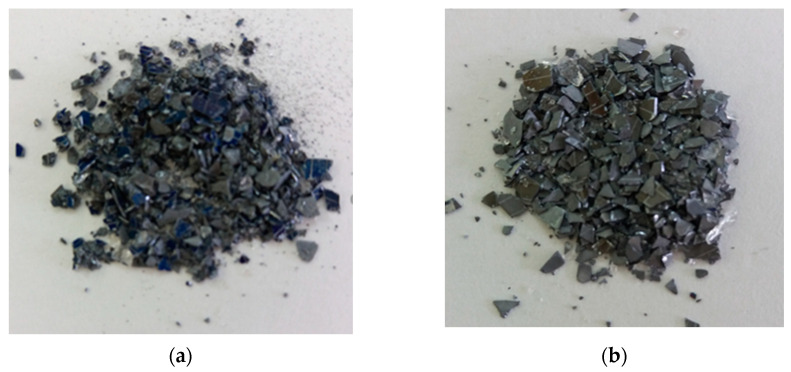
Photograph of recovered silicon after (**a**) 1.5 h etching time (**b**) 24 h etching time.

The appearance of the recovered particles after chemical treatment is shown in Figure 2. The particles’ dimensions were in the range of hundreds of micrometers to one millimeter. In Figure 2a it is observed that the surface of some of these particles was blue, which suggests the presence of the antireflection layer. As the etching time increased, this coloration was no longer visible on the surfaces of the particles (Figure 2b).

**Figure 3:**
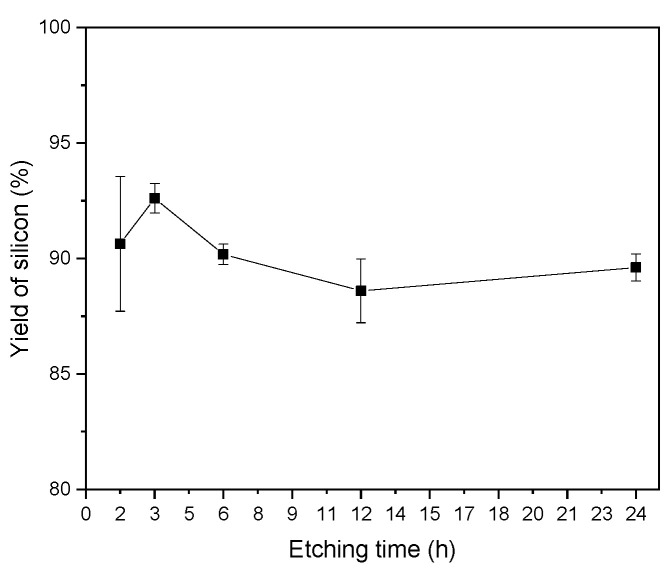
Yields of the silicon as a function of etching time.

The fractions of recovered silicon were weighted and their mass percentage considering the initial amount in the sandwich before the chemical treatment was calculated. This mass percentage of the recovered solid as a function of the treatment time is shown in Figure 3. Each point represented in the graph indicates the yield calculation in three groups of samples treated in the corresponding time interval.

Approximately 90% of the solid that underwent chemical treatment was recovered regardless of the treatment time. The divergence observed in the error bars was believed to be caused by the glass fragments present in some samples.

**Figure 4:**
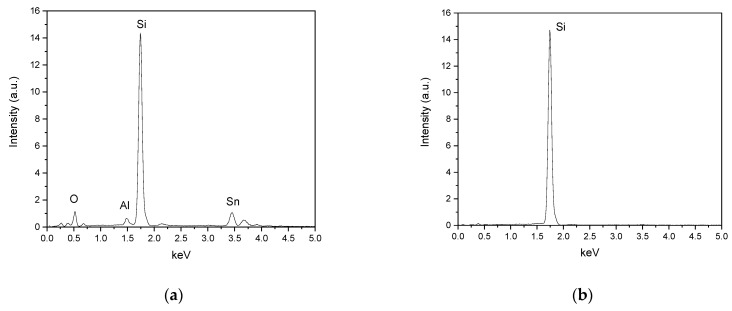

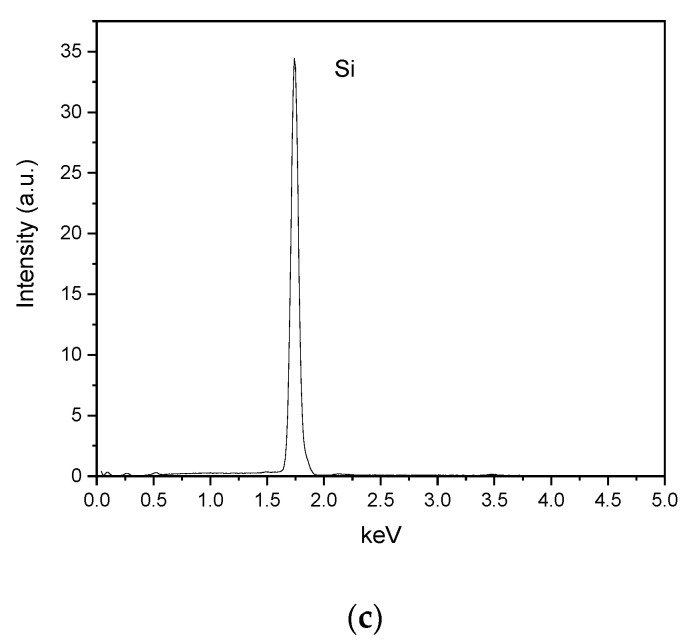
EDX spectra of silicon wafer particles recovered at (**a**) 1.5 h (**b**) 6 h and (**c**) 24 h etching time.

Different particles from samples treated at 1.5, 6, and 24 h were analyzed by Energy Dispersive X-ray Spectroscopy. We observed that the X-ray photon peaks corresponding to Cu, and Pb were not shown in the spectra indicating that its concentration was below the detection limit of the EDS technique. The composition analysis after different immersion times showed the presence of elements such as Ag and Sn in samples recovered after 1.5 h of treatments (Figure 4a). The typical spectra of the samples immersed for a longer time in the etching solution show the reduction of impurity in silicon samples (Figure 4b,c). A deeper analysis would have to be done to determine the composition of the samples at every stage during the etching processes.

**Figure 5:**
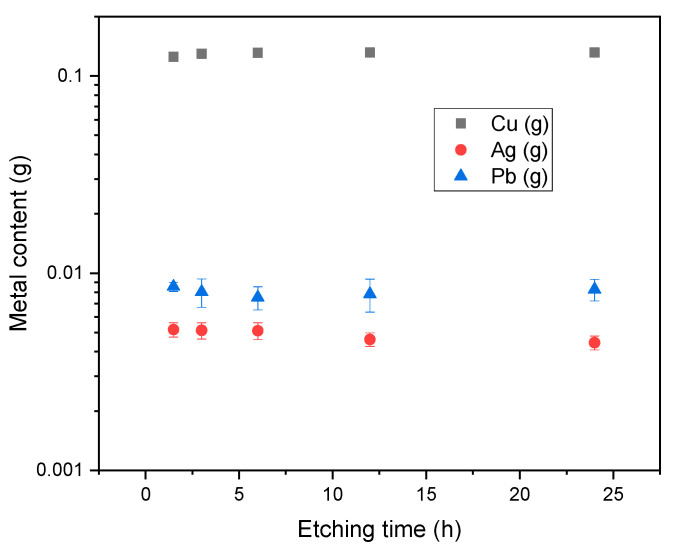
Influence of etching time on dissolved metal content.

Regarding the metals of interest in this work (Cu, Ag, Pb), they were obtained after the chemical treatment in the form of ions (Cu2^+^, Pb^2+^, Ag^+^) in the solution. The quantification of these metals was also done as a function of etching time, using the Atomic Absorption Spectroscopy technique (see Figure 5).

The most abundant metal in the solution was copper because it is the primary material for metallic contacts, due to the combination of both its conductivity and price. Lead, which is present in the busbars as a coating, was found in smaller quantities along with silver which is present in the rear side metallization and/or front side electrode of solar cells. Statistical analysis of data exhibited in Figure 3 and Figure 5, shows that both the amount of silicon and other metals recovered was not influenced by etching time. A time of 1 h was enough to transfer Ag, Cu, Pb into the solution.

After these experiments we conclude that (1) both the amount of recovered silicon and the concentration of Cu, Ag, and Pb in the solution were not affected by the immersion time in the solution, (2) the removal of the anti-reflective layer was achieved when the samples were immersed for long times, (3) a semi-qualitative analysis suggested that quality of recovered silicon increases after 3 h of treatment. Therefore, 24 h was established as a time of immersion in the solution for further experiments to ensure the complete removal of the AR coating.

#### 3.2.2. Metal Content Analysis

The optimal treatment conditions determined previously were applied to three samples of each selected panel. Following the same procedure, the average weigh percentages of recovered silicon were calculated for each group. Experimental results denoted a similar percentage for the different kinds of panels resulting in 89 and 94% for the polycrystalline panels, P1, P2, and 91% for monocrystalline panel P3. It should be noted that the size of the recovered silicon particles was not homogeneous, and, in some cases, they were still attached to glass fraction. This problem has been studied before and can be solved by taking advantage of the different properties of the two materials [28]. The smaller particles contained more silicon, so, as larger particles were removed, the quality of the recovered silicon increased, and the average weight percentages decreased.

The filtered solutions were analyzed using AAS to quantify metals concentration for each sample group. The results and the corresponding statistical analyses are shown in Table 1.

The data available in the literature about the metal content in PV panels slightly differ because they depend on some aspects as the fraction of the panel considered for calculation, the materials composition used by the specific manufacturer, and the year of panel fabrication [29]. According to Dominguez at el, the average percentage of Cu, Pb, and Ag in silicon modules are 7.3 × 10^−1^%, 4.6 × 10^−3^%, and 5.7 × 10^−2^% respectively [22]. Our results show that the Cu content is the highest in the three studied samples, as expected. The content of the other metals, Ag and Pb, were at least an order of magnitude lower than Cu, in all samples. In the polycrystalline panels, (P1, P2) was detected a greater amount of lead than silver. However, the Ag content in the monocrystalline panel (P3), was almost five times greater than the quantity found in the two polycrystalline ones. As we know the silver is used as electrode in the front side and the metallization in the rear side in silicon PV panels. We relate the difference in the silver content found in this work, to the differences in the manufacturing processes of these panels. The recovery of dissolved metals in the solution can be achieved by using different methods [14] but this study was not included in the aim of this work.

## 4. Conclusions

The conditions of thermal and chemical treatment were optimized to separate metals and recover silicon from damaged PV panels. The thermal method was applied to remove EVA. The explored factors for this step were time interval and temperature that would ensure minimum energy expenditure and complete combustion of samples. These conditions were fulfilled by heating the samples at 650 °C for 30 min.

After delamination was investigated the effect of immersion time on the separation efficiency of silicon and metals during the etching process using a mixture of 5 mL of HNO3 (70%) and 0.5 mL of HF (40%), in a 10: 1 proportion. The percentage mass of recovered silicon and dissolved metal content were similar for tested immersion times. However, the particle appearance changed as time became longer suggesting anti-reflective layer removal from the silicon surface. A deeper analysis must be done to determine the purity of the recovered samples. Two polycrystalline and one monocrystalline panel were treated with the optimized parameters found for the thermal and chemical method. According to the experimental results, a similar amount of silicon can be recovered, regardless of tested PV technology. In addition, the concentration of Cu and Pb in the solution is similar for the three panels, but the Ag content appears in higher concentrations in monocrystalline one which is related to different fabrication technology. The results confirm the usefulness of the optimized methodology applied to PV damaged modules for silicon recovery and metal separation. As far as we know this work is one of the first steps for the development of a recycling photovoltaic process in Mexico. Additional studies are currently performed to minimize environmental contamination due to the emission of toxic gases during the process and green chemical treatments for the pH neutralization of residual solutions.

## Figures and Tables

**Table 1:** Results from the AAS for P1, P2, and P3 samples.

Samples	Cu(g)	Ag(g)	Pb(g)
P1	0.1281 ± 0.0062	0.0043 ± 0.0002	0.0085 ± 0.0016
P2	0.1232 ± 0.0016	0.0041 ± 0.0011	0.0060 ± 0.0012
P3	0.1291 ± 0.0020	0.0199 ± 0.0021 *	0.0059 ± 0.0012

* statistically significant difference (*p* < 0.05).

## Data Availability

The data presented in this study are available on request from the corresponding author.
